# Expression of Concern: Mitochondrial dynamic modulation exerts cardiometabolic protection in obese insulin-resistant rats

**DOI:** 10.1042/CS20190960_EOC

**Published:** 2025-06-19

The Editorial Office has been made aware of potential issues surrounding the scientific validity of this paper and would therefore like to issue an Expression of Concern. It has been noted that there may be incidences of splicing and similarities between western blots within this manuscript. Specifically, there were concerns over potential splice sites in the Figure 3D Cleaved caspase 3 row, and in the Figure 6F Total Drp1, Mfn2 and OPA1 rows. There also appear to be similarities between the last bands in the Figure 6F Mito-Drp1 and Total Drp1 rows. The authors provided raw data and disagreed with all splicing and similarities found. The Journal will therefore keep an Expression of Concern published to notify readers, but no further action will be taken at this time

